# Speech-evoked activation in adult temporal cortex measured using functional near-infrared spectroscopy (fNIRS): Are the measurements reliable?

**DOI:** 10.1016/j.heares.2016.07.007

**Published:** 2016-09

**Authors:** Ian M. Wiggins, Carly A. Anderson, Pádraig T. Kitterick, Douglas E.H. Hartley

**Affiliations:** a National Institute for Health Research (NIHR) Nottingham Hearing Biomedical Research Unit, 113 The Ropewalk, Nottingham, NG1 5DU, United Kingdom; b Otology and Hearing Group, Division of Clinical Neuroscience, School of Medicine, University of Nottingham, Nottingham, NG7 2UH, United Kingdom; c Medical Research Council (MRC) Institute of Hearing Research, University of Nottingham, University Park, Nottingham, NG7 2RD, United Kingdom; d Nottingham University Hospitals NHS Trust, Derby Road, Nottingham, NG7 2UH, United Kingdom

**Keywords:** Auditory cortex, fNIRS, Functional near-infrared spectroscopy, Speech, Speechreading, Test-retest reliability

## Abstract

Functional near-infrared spectroscopy (fNIRS) is a silent, non-invasive neuroimaging technique that is potentially well suited to auditory research. However, the reliability of auditory-evoked activation measured using fNIRS is largely unknown. The present study investigated the test-retest reliability of speech-evoked fNIRS responses in normally-hearing adults. Seventeen participants underwent fNIRS imaging in two sessions separated by three months. In a block design, participants were presented with auditory speech, visual speech (silent speechreading), and audiovisual speech conditions. Optode arrays were placed bilaterally over the temporal lobes, targeting auditory brain regions. A range of established metrics was used to quantify the reproducibility of cortical activation patterns, as well as the amplitude and time course of the haemodynamic response within predefined regions of interest. The use of a signal processing algorithm designed to reduce the influence of systemic physiological signals was found to be crucial to achieving reliable detection of significant activation at the group level. For auditory speech (with or without visual cues), reliability was good to excellent at the group level, but highly variable among individuals. Temporal-lobe activation in response to visual speech was less reliable, especially in the right hemisphere. Consistent with previous reports, fNIRS reliability was improved by averaging across a small number of channels overlying a cortical region of interest. Overall, the present results confirm that fNIRS can measure speech-evoked auditory responses in adults that are highly reliable at the group level, and indicate that signal processing to reduce physiological noise may substantially improve the reliability of fNIRS measurements.

## Highlights

• First study to assess the reliability of speech-evoked fNIRS responses in adults.
• Responses to auditory speech were highly reliable at the group level.
• Temporal-lobe responses to visual speech were less reliable.
• Reliability at single-subject level varied widely among individuals.
• Signal processing to reduce physiological noise improved reliability at group level.

## 1 Introduction

Functional near-infrared spectroscopy (fNIRS) has emerged as a popular method for imaging the haemodynamic response to neuronal activity in the human brain (Boas et al., 2014). This non-invasive technique uses near-infrared light to illuminate the brain through the intact scalp; the intensity of light returning to the surface is measured to detect changes in cerebral haemoglobin concentrations. Changes in the concentration of oxygenated (HbO) and de-oxygenated (HbR) haemoglobin are used as indicators of cortical activation, based on the tight coupling that exists between neuronal activity and oxygen delivery (Iadecola, 2004). The technique has been used extensively to study language processing (for a review, see Quaresima et al., 2012), and in recent years interest has grown in using fNIRS to study central auditory processing directly (Abla and Okanoya, 2008, Chen et al., 2015, Lawler et al., 2015, Plichta et al., 2011, Pollonini et al., 2014, Sevy et al., 2010). Certainly, fNIRS has practical characteristics that are well suited to auditory research: not only is it portable and relatively inexpensive, it is quiet, requires a low degree of participant tolerance, and is suitable for imaging patients with magnetic implants. Thus, unlike functional magnetic resonance imaging (fMRI), fNIRS is free from the issues posed by acoustic scanner noise (Peelle, 2014), is ideally suited for developmental studies in infants (Lloyd-Fox et al., 2010), and is compatible with implanted auditory prostheses, e.g. cochlear implants (Sevy et al., 2010).

However, for fNIRS to become an accepted tool in auditory research, it is critical to establish the fundamental capabilities of the technique, including its ability to measure auditory responses that are reproducible across test sessions. Adequate test-retest reliability is the foundation of successful neuroimaging research, and is especially pertinent where prospective longitudinal studies are concerned (Blasi et al., 2014, Lawler et al., 2015): only if measurements are sufficiently reliable will it be possible to identify changes in cortical activation associated with (ab)normal development or clinical intervention. Subsequently, the present study aimed to quantify the test-retest reliability of temporal-lobe fNIRS responses to auditory speech (with and without matching visual cues) in normally-hearing adults. Since visual speech cues are also known to activate auditory regions in the superior temporal cortex (Calvert et al., 1997, Hall et al., 2005, MacSweeney et al., 2000), we additionally aimed to quantify the reliability of temporal-lobe responses to visual speech (i.e. silent speechreading).

The test-retest reliability of fNIRS in adults has previously been studied outside of the auditory domain. Here, we restrict our focus to studies that assessed the reliability of functional activation in response to a defined stimulus or task. Using metrics previously established in the fMRI test-retest literature (e.g. Aron et al., 2006, Johnstone et al., 2005, Manoach et al., 2001, Rombouts et al., 1997), these studies typically evaluated, across test sessions and in varying combinations, the spatial reproducibility of cortical activation patterns, similarity in the shape of the measured haemodynamic time courses, and the consistency of response amplitude (typically quantified using the intraclass correlation coefficient, ICC). In one of the first of these studies, Watanabe et al. (2003) concluded that fNIRS offered acceptable reliability for measuring frontal cortical activation in response to cognitive tasks, based on results from a small sample of five subjects. Subsequent studies (Kakimoto et al., 2009, Kono et al., 2007, Schecklmann et al., 2008) confirmed the capability of fNIRS to reliably measure prefrontal activation during cognitive tasks, at least when responses are considered at the group level and when averaged across multiple channels overlying a cortical region of interest (ROI). Variable, and generally poorer, reliability has been observed at single-subject and single-channel level, leading Schecklmann et al. (2008) to conclude that such analyses should be interpreted with caution.

Similar findings have emerged from fNIRS test-retest studies that focused on other cortical regions. In the sensorimotor cortex, the time course of activation in response to simple motor tasks has been found to be reproducible within subjects (Pearson's *r* > 0.6) over intervals of minutes (Strangman et al., 2006) and months (Sato et al., 2006). In a comprehensive assessment of the reliability of sensorimotor activation during an event-related finger-tapping paradigm, Plichta et al. (2007b) found fNIRS-measured activation to be highly reproducible at the group level across a retest interval of three weeks; however, they cautioned that at the single-subject level reproducibility metrics generally did not exceed “low to mediocre” values. Similarly, in the visual domain, occipital activation in response to periodic checkerboard stimulation was found to have good-to-excellent reproducibility at the group level, while reliability at single-subject level was variable among individuals and only “low” to “moderate” on average (Plichta et al., 2006).

To our knowledge, only one assessment of fNIRS test-retest reliability to auditory stimulation has been published to date (Blasi et al., 2014). This study was conducted in infants aged 4–16 months and used a retest interval of 8.5 months. The authors based their analyses primarily on the HbO parameter, which was found to be the more robust of the two haemoglobin chromophores (HbO and HbR). In response to human vocal sounds (e.g. yawning, crying, laughing) versus silence, at the group level the authors found excellent reproducibility of i) the spatial pattern of right temporal activation (overlap across sessions up to 94%), and ii) the shape of the haemodynamic time course (Pearson's *r* = 0.90). However, in the same study, response amplitude was less reliable across test sessions (ICCs mostly below 0.5). Also, consistent with the adult studies described above, reliability was highly variable for individual infants. While Blasi et al.’s study confirms that fNIRS is capable of measuring reliable auditory responses in infants (at least at the group level), the findings cannot be directly extrapolated to adults because of differences in light propagation between infant and adult heads (Fukui et al., 2003), as well as the challenge of separating the reliability of the measurement technique from potential developmental changes that may occur in these infants over the same time period.

A prevalent issue in fNIRS imaging is that the measurements are more sensitive to absorption changes occurring in superficial tissue layers than in deeper structures such as the cortex (Strangman et al., 2013). This makes the measurements highly susceptible to interference from physiological signals of extra-cerebral origin (Huppert et al., 2009). One promising approach to minimize the influence of superficial signals is to introduce a reference channel with short (ideally <10 mm) source–detector separation: this allows signals that originate in superficial tissue layers (i.e. the scalp and skull) to be regressed out from the main measurement channels (Gagnon et al., 2011, Saager and Berger, 2005). However, a disadvantage of this approach is that the hardware used for data acquisition must provide for the additional short-separation measurement channels, something that is not true of many commercially available fNIRS systems. An alternative approach, which does not require any modification of the acquisition system, was proposed by Yamada et al. (2012). Yamada et al. described a signal-processing algorithm that exploits the fact that changes in HbO and HbR tend to be negatively correlated in the functional cerebral response, whereas systemic physiology tends to give rise to positively correlated changes in HbO and HbR. On this basis, the algorithm aims to separate the haemodynamic signal into estimates of the functional and systemic components. We have found application of this algorithm to be beneficial in earlier work (Wiggins and Hartley, 2015), and here we aimed to establish whether it can improve the test-retest reliability of fNIRS measurements, compared to the conventional approach of assessing HbO and HbR separately.

Based upon the findings of the previous research, our overall predictions were as follows: i) that speech-evoked activation in adult temporal cortex measured using fNIRS would be more reliable at group and multi-channel level, compared to single-subject and single-channel level; and ii) that application of Yamada et al.'s (2012) algorithm to isolate the functional component of the haemodynamic signal would improve test-retest reliability.

## 2 Materials and methods

### 2.1 Participants and test sessions

Seventeen participants (median age 65 years, range 26–75 years, 6 males) were tested in two sessions separated by approximately 3 months (median number of days between sessions 91, range 85–108 days). All participants had normal hearing as assessed using pure-tone audiometry conducted during session 1 (average air-conduction threshold ≤20 dB HL across frequencies 0.5, 1, 2 and 4 kHz in both ears). All participants were native English speakers with normal or corrected-to-normal vision and no known cognitive or motor disorders. Most participants (*N* = 15) were right-handed as assessed using the Edinburgh Handedness Inventory (Oldfield, 1971). The study was approved by the Nottingham 1 Research Ethics Committee and all participants gave written informed consent.

### 2.2 Test procedure

In a block design, participants were presented with 24-s blocks of speech stimulation interleaved with rest periods of random duration in the range 20–40 s. There were three stimulation conditions, including an auditory-only (A-ONLY), visual-only (i.e. silent speechreading; V-ONLY), and audiovisual (AV) condition. In the A-ONLY condition, the background remained uniform and a fixation cross was presented at the position where the talker's mouth would have been. This uniform background and fixation cross remained visible throughout rest periods also. The stimulation conditions were interleaved in random order, with each condition presented five times in total. The overall measurement duration was approximately 15 min.

Participants were instructed to look at the fixation cross during rest periods. During stimulation periods, participants were instructed to attend to the speech and to try to understand what was said. Although, for the most part, there was no active task, to encourage sustained attention to the experimental stimuli infrequent control trials were presented (after two of the 15 stimulation blocks, chosen at random). The control trials proceeded as follows: Two seconds after the cessation of a chosen block, two words were presented on either side of the fixation cross; in a two-alternative forced-choice task, participants were asked to press one of two buttons to indicate which of the two words had been spoken in the immediately preceding sentence. The response period was set to automatically time out if no button press was registered within 15 s, although participants were observed to always respond within this period. Following the response, the ensuing rest period was extended by 5 s. Note that responses to these control trials were not examined as part of the data analysis.

Before commencing data collection, there was a short familiarization session in which each of the three stimulation conditions was presented once, in each case followed by a practice of the control task. This familiarization session was conducted before the optode array was placed on the participant's head. Immediately after the fNIRS measurements, a test of speech intelligibility was conducted to assess participants' ability to correctly report keywords (three per sentence) in each of the three stimulation conditions.

### 2.3 Stimuli and equipment

**Fig. 1:**
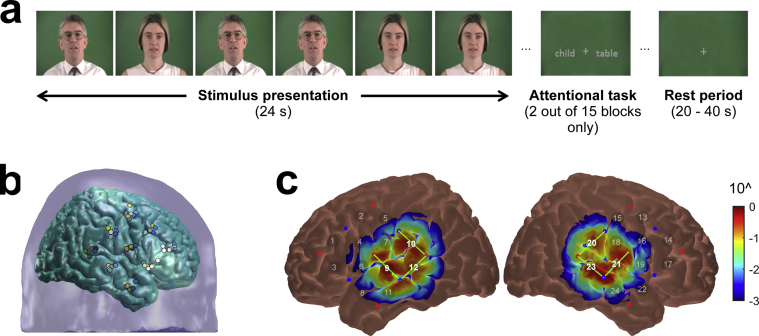
(a) Schematic representation of one cycle of the block-design stimulus presentation paradigm; (b) Variability in optode positioning across six volunteers after registration to a standard atlas brain. Optode positions for each volunteer are represented by different coloured dots. Variability was similar in the left hemisphere (data not shown); (c) Aggregate sensitivity profiles for the predefined auditory regions-of-interest, which comprised the three highlighted measurement channels in each hemisphere. The colour scale depicts relative sensitivity logarithmically from 0.001 to 1.

We used the IHR Number Sentences developed by Hall et al. (2005), which include sentences such as “Five yellow leaves are falling.” Digital audiovisual recordings of 90 such sentences were used, each spoken by both a male and a female talker. These 90 sentences were randomly split into sets of 30, one set for each of the three conditions (A-ONLY, V-ONLY, and AV). Each stimulation block comprised six concatenated sentences (three from each talker), presented in random order and evenly spaced to fill the 24-s block (Fig. 1a). To minimize familiarity effects, for each participant a different random allocation of sentences to conditions, as well as a different grouping of sentences into blocks, was used at each session. The same sentences were used for the speech intelligibility tests conducted immediately after fNIRS imaging, again with a different allocation of sentences to conditions; furthermore, the talker gender was reversed, such that sentences that were spoken by the female talker during fNIRS imaging were spoken by the male talker during the speech intelligibility tests, and vice versa.

Testing was conducted in a soundproof booth with dimmed lighting. Participants were seated comfortably at a distance of 75 cm from the visual display. Auditory stimuli were presented through a Genelec 8030A loudspeaker mounted immediately above and behind the display, at a level of 65 dB SPL (A-weighted root-mean-square level averaged over the duration of each sentence, measured at the listening position using a Brüel & Kjær Type 2250 sound level meter with the participant absent). Participants responded to the control trials using a response box held on their lap. A dense sound-absorbing screen was placed between the fNIRS equipment and the listening position, resulting in a steady ambient noise level of 38 dB SPL (A-weighted).

### 2.4 fNIRS measurements

Measurements were made using a continuous-wave fNIRS system (ETG-4000, Hitachi Medical Co., Japan). The probe set comprised two 3 × 3 arrays (each containing 5 sources and 4 detectors), which were used to simultaneously record responses from the left and right cerebral hemispheres. This gave 24 measurement channels in total, with a fixed source-detector spacing of 30 mm. The ETG-4000 measures simultaneously at wavelengths of 695 nm and 830 nm (sampling rate 10 Hz), and uses frequency modulation to minimize crosstalk between wavelengths and optodes.

The probe set was positioned on the head so as to ensure good coverage of superior temporal regions, where the auditory cortices are located (Penhune et al., 1996). To allow for a valid assessment of fNIRS test-retest reliability, it was important to ensure that the optodes were positioned as consistently as possible across participants and test sessions. The international 10–20 system (Jasper, 1958) was therefore used to guide optode placement: on each side, the lowermost source optode was placed as close as possible to the preauricular point, with the uppermost source optode aligned towards position Cz. Consistency of positioning across test sessions at the individual level was further ensured by reference to photographs taken during session 1. Once the position of the probe set was finalized, an elasticised bandage was gently wrapped around the participant's head to help maintain secure contact between the optodes and the scalp. Participants were asked to sit as still as possible during testing to minimize motion artefacts.

To evaluate the consistency of positioning across individuals, the procedure was piloted on six adult volunteers who did not take part in the main study. After positioning the probe set as described above, the optode positions, plus anatomical surface landmarks, were recorded using a 3D digitizer. These were then registered to the “Colin27” atlas brain (Collins et al., 1998) using the AtlasViewer tool (Aasted et al., 2015). Fig. 1b gives an indication of the spread in registered optode positions across the six volunteers. The standard deviation in the position of each optode was between 2.9 and 8.8 mm. We did not expect this degree of variability to have a pronounced effect on our recordings, given that the spatial resolution of our non-overlapping fNIRS measurements was expected to be comparable to the 30-mm source-detector spacing (Boas et al., 2004).

**Fig. S1:**
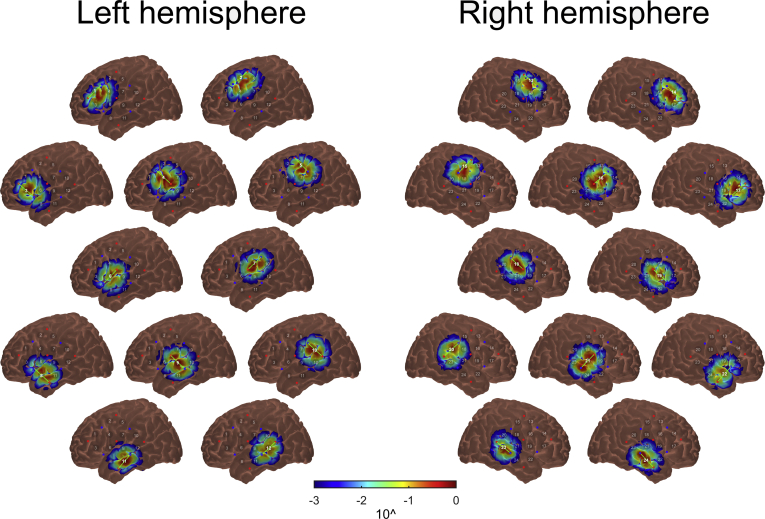
Sensitivity profiles for individual fNIRS measurement channels. The colour scale depicts relative sensitivity logarithmically from 0.001 to 1.

We also used AtlasViewer to confirm that our probe set provided sensitivity to the relevant cortical regions. The software calculates a cortical sensitivity profile for each measurement channel by running the photon migration forward problem, i.e. by simulating the probabilistic path of photons as they traverse through the head from source to detector (Aasted et al., 2015). The forward problem was run using the Monte-Carlo photon transport software tMCimg (Boas et al., 2002), with 1 × 10^7^ simulated photons launched from each optode. Based on the anatomical registrations reported above, alongside published results obtained using the same imaging system (Plichta et al., 2011) and our own piloting data, we predefined two ROIs in which we expected to observe significant auditory cortical activation. These ROIs, located over left and right posterior superior temporal regions, each comprised three measurement channels (left hemisphere: Ch#s 9, 10, 12; right hemisphere: Ch#s 20, 21, 23). The selection of three channels in each hemisphere, rather than more or fewer channels, was considered an appropriate compromise to ensure adequate coverage of the relevant cortical regions while also ensuring that the ROIs remained focused only on areas that were expected to be significantly activated. Fig. 1c shows the aggregate sensitivity profiles for the ROIs, which confirm that the measurements provided good sensitivity to the posterior superior temporal gyri as well as surrounding areas. For reference, the sensitivity profiles of all individual measurement channels are included as Supplementary Fig. S1.

### 2.5 Data analysis

Data analysis was performed in MATLAB (MathWorks, Natick, MA) using functions provided in the HOMER2 package (Huppert et al., 2009) together with custom scripts.

#### 2.5.1 Pre-processing

The raw fNIRS recordings were pre-processed to reduce the influence of motion artefacts and physiological noise before estimating changes in the concentration of HbO and HbR. First, the raw intensity signals were converted to changes in optical density (Huppert et al., 2009). Correction for motion artefacts was then performed using wavelet filtering, which has emerged as a promising approach to dealing with motion artefacts in fNIRS recordings (Brigadoi et al., 2014, Cooper et al., 2012). We used a simplified form of the algorithm described by Molavi and Dumont (2012), as implemented in the HOMER2 *hmrMotionCorrectWavelet* function. This function applies a probability threshold to remove outlying wavelet coefficients, which are assumed to correspond to motion artefacts. We excluded any coefficients lying more than 1.5 inter-quartile ranges below the first quartile or above the third quartile.

Following motion-artefact correction, the optical density signals were band-pass filtered between 0.01 and 0.5 Hz to attenuate low-frequency drift and cardiac oscillations, and then converted into estimates of changes in the concentration of HbO and HbR using the modified Beer-Lambert law (Huppert et al., 2009). We used a default value of 6 for the differential path-length factor at both wavelengths. Since we did not account for the partial volume effect associated with focal cortical activation, estimates of absolute changes in haemoglobin concentrations are subject to considerable uncertainty and should not be interpreted directly (Boas et al., 2001). Relative response magnitudes remain interpretable, however, and form the basis of our assessment.

#### 2.5.2 Haemodynamic measures

Changes in HbO and HbR concentration were separately assessed as indicators of cortical activity, as is customary in the fNIRS literature. In addition, we assessed a third haemodynamic measure: the estimated functional component of the haemodynamic response, derived using the haemodynamic modality separation (HMS) algorithm described by Yamada et al. (2012). This algorithm aims to reduce the influence of systemic physiological signals by exploiting the fact that ΔHbO and ΔHbR are negatively correlated in the functional cerebral response, while systemic physiology (and head motion) tends to give rise to positively correlated changes in HbO and HbR (Cui et al., 2010, Yamada et al., 2012). Briefly, the fNIRS signal is modelled as a mixture of a functional component, in which a fixed negative linear relationship is assumed between ΔHbO and ΔHbR, and a systemic component, in which a positive linear relationship is assumed between ΔHbO and ΔHbR. The strength of the relationship between ΔHbO and ΔHbR in the systemic component is expected to be task-dependent and is therefore estimated from the data by minimizing the mutual information between the functional and systemic components. The HMS algorithm returns separate estimates of the functional and systemic components; we extracted ΔHbO for the functional component for further analysis.

#### 2.5.3 Statistical analysis

To quantify the amplitude of the haemodynamic response and test for significant cortical activation we performed statistical analyses based on the general linear model (GLM). We used a two-stage ordinary least squares (OLS) estimation procedure similar to that employed by Plichta et al., 2006, Plichta et al., 2007b. The design matrix included three boxcar regressors (one for each stimulation condition), plus an additional regressor-of-no-interest comprising delta functions indicating the onsets of the control trials, all convolved with the canonical haemodynamic response function (HRF) provided in SPM8 [http://www.fil.ion.ucl.ac.uk/spm]. We selected a parsimonious model that did not include any temporal derivatives of the HRF, since preliminary analyses indicated no benefit of including the derivative terms for the present dataset. After completing the first-stage OLS estimation at the single-subject level, we used the Cochrane-Orcutt procedure to correct for serial correlation (Cochrane and Orcutt, 1949). Briefly, this involved fitting a first-order autoregressive process to the model residuals and transforming the original model according to the estimated autoregressive parameter (see Plichta et al., 2007a). We then re-estimated the beta weights based on the transformed model (second stage). The beta weights, which represent the amplitude of the haemodynamic response, formed the parameter set for subsequent hypothesis testing. We tested for significant cortical activation in each stimulation condition (compared to rest) by using one-sided *t*-tests to compare the relevant beta weight to zero (alpha level 0.05); cortical activation is indicated by positive *t*-values for the estimated functional component and HbO, and by negative *t*-values for HbR. We tested for significant cortical activation both at single-subject level and in a group-level random-effects analysis.

To account for the multiple comparisons issue posed by separately testing for significant activation in each channel, we used the false discovery rate (FDR) method (Benjamini and Hochberg, 1995, Benjamini and Yekutieli, 2001). The application of FDR methods in fNIRS was investigated in detail by Singh and Dan (2006), and the approach has been adopted in several previous assessments of fNIRS test-retest reliability (Blasi et al., 2014, Kakimoto et al., 2009, Kono et al., 2007, Schecklmann et al., 2008). We used the more conservative version of the FDR procedure described by Benjamini and Yekutieli (2001), since this did not require us to make any assumptions about the pattern of dependencies between fNIRS channels.

### 2.6 Tests for reliability

We used established metrics to assess the reliability of cortical responses at both group and single-subject level. Specifically, we aimed to quantify:

(1) **Reproducibility in the pattern of cortical activation elicited by auditory, visual, and audiovisual speech.** Reproducibility of the activation pattern across the entire probe set was assessed by calculating the linear correlation between the individual-channel *t*-values at sessions 1 and 2 (Plichta et al., 2006, Plichta et al., 2007b). For a more focused view, we calculated the reproducibility of the quantity (*R*_QUANTITY_) and location (*R*_OVERLAP_) of significantly activated channels (Blasi et al., 2014, Kono et al., 2007, Plichta et al., 2006, Plichta et al., 2007b, Rombouts et al., 1997, Schecklmann et al., 2008). We used the following definitions:

$$\begin{array}{l} R_{\mathrm{QUANTITY}}=1-\left|A_{1}-A_{2}\right|/\left(A_{1}+A_{2}\right)\text{,}\\ R_{\mathrm{OVERLAP}}=2\;x\;A_{\mathrm{OVERLAP}}/\left(A_{1}+A_{2}\right)\text{,} \end{array}$$

where A_1_ is the number of significantly activated channels at session 1, A_2_ is the number of significantly activated channels at session 2, and A_OVERLAP_ is the number of identical channels that showed significant activation at both sessions. *R*_QUANTITY_ and *R*_OVERLAP_ take values between 0 (indicating no reproducibility) and 1 (indicating perfect reproducibility).

(2) **Reliability of cortical responses within the ROIs.** To quantify the reliability of response amplitude, we used the ICC. Conceptually, the ICC represents the proportion of the total variance that can be attributed to between-subject differences. That is:

$$ICC=\frac{Between-subjects\;variance}{Between-subjects\;variance+Between-sessions\;variance}$$

ICCs close to 1 represent high reliability and occur when the between-subjects variance is much larger than the between-sessions variance (i.e. the within-subject variance across repeated measurements) (Johnstone et al., 2005); ICCs close to 0 represent poor reliability. Negative ICCs, which can occur due to sampling uncertainty, but which have no theoretical meaning, were replaced by 0 (Li et al., 2015). In practice, the ICC is derived from an appropriate analysis of variance (ANOVA) (see Li et al., 2015 for details of the calculation). Like previous studies that sought to quantify the test-retest reliability of fNIRS (Blasi et al., 2014, Kakimoto et al., 2009, Plichta et al., 2006, Plichta et al., 2007b, Schecklmann et al., 2008), we used the one-way random-effects model (Shrout and Fleiss, 1979). Within each ROI, we calculated ICCs both for the constituent channels (“single-channel ICCs”) and after averaging across channels (“cluster-level ICCs”). Two types of ICC were calculated: ICC_SINGLE_SESS_, which provides an indication of the reliability of measurements made at a single session, and ICC_2_SESS_AVG_, which indicates the reliability of the mean response across the two sessions (Johnstone et al., 2005). Additionally, we tested for any significant differences in mean response amplitude between sessions using paired *t*-tests, reporting in each case an effect-size estimate based on Cohen's *d* for paired observations (Cohen, 1988). Finally, we assessed reproducibility in the shape of the haemodynamic response by calculating the linear correlation between the block-averaged time courses of haemoglobin concentration changes at sessions 1 and 2 (Blasi et al., 2014, Kakimoto et al., 2009, Kono et al., 2007, Sato et al., 2006, Schecklmann et al., 2008).

To guide interpretation of the results, we assessed the various reliability metrics (linear correlation coefficients, ICCs, *R*_QUANTITY_ and *R*_OVERLAP_) against the following criteria put forward for ICCs by Li et al. (2015): poor (<0.40), fair (0.40–0.59), good (0.60–0.74), excellent (0.75–1.00). These criteria are broadly comparable to the cut-off values adopted in previous studies of fNIRS and fMRI test-retest reliability (Aron et al., 2006, Blasi et al., 2014, Manoach et al., 2001, Plichta et al., 2006, Plichta et al., 2007b, Schecklmann et al., 2008). In assessing effect sizes based on Cohen's *d*, we adopted Cohen's (1988) guideline criteria for small (0.20–0.49), medium (0.50–0.79), and large (≥0.8) effects.

## 3 Results and discussion

### 3.1 Speech intelligibility

**Fig. 2:**
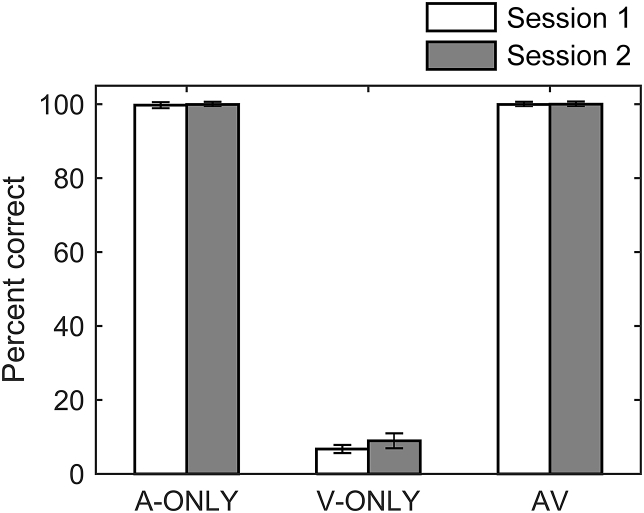
Mean percentage of correctly reported keywords in a test of speech intelligibility conducted immediately after the fNIRS measurements and based on the same speech corpus. Error bars show ±1 standard error of the mean, corrected to account for the repeated-measures design (Field, 2009).

The speech intelligibility data (Fig. 2) collected immediately after the fNIRS measurements confirmed that participants were readily able to understand the auditory speech material with or without visual cues (mean percentage of correctly reported keywords ≈100% in the A-ONLY and AV conditions). In contrast, speech intelligibility based on visual cues alone was uniformly poor (V-ONLY condition, mean scores <10%). Similarly low levels of speechreading performance by normally-hearing individuals when tested on open-set sentence recognition tasks have been reported in the literature (Altieri et al., 2011, Stacey et al., 2016). Thus, in interpreting the following results, it is important to note that fNIRS test-retest reliability in the A-ONLY and AV conditions reflects a response to highly intelligible speech, while reliability in the V-ONLY condition reflects a response to a largely unintelligible stimulus. Mean intelligibility scores were numerically similar at sessions 1 and 2 (within 2.2%-points), suggesting that the speech material was approximately equally intelligible at the two sessions.

### 3.2 Reproducibility of cortical activation patterns

#### 3.2.1 Group level

**Fig. 3:**
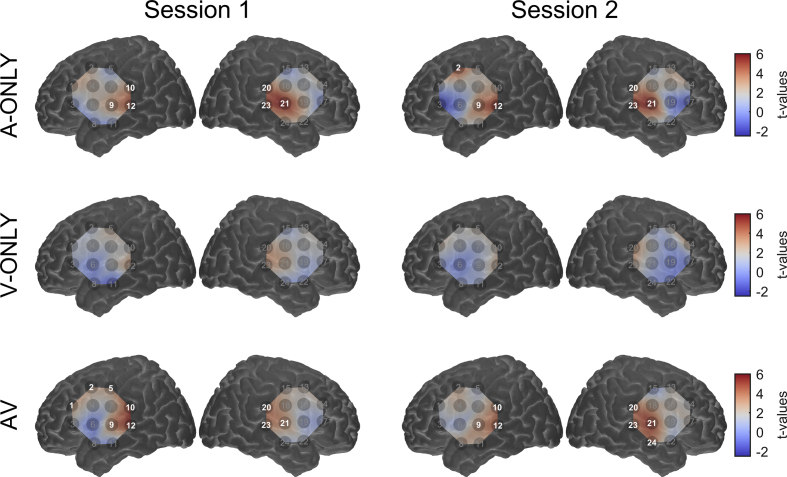
Group-level activation maps overlaid on a standard brain. Results for each stimulation condition are shown in a separate row. Channels that showed significant activation are highlighted (*p* < 0.05 after false discovery rate correction). Note that the maps are interpolated from single-channel results and the overlay on the cortical surface is for illustrative purposes only. This figure is for the estimated functional component of the haemodynamic response; for corresponding figures for HbO and HbR assessed separately, see Supplementary Figs. S2 and S3.

Focusing on the results for the estimated functional component of the haemodynamic response, we were able to reliably detect significant group-level activation to auditory speech (with or without visual cues) in and around the predefined ROIs over the left and right superior temporal gyri (Fig. 3, A-ONLY and AV conditions). This confirms the capability of fNIRS to detect group-level auditory cortical activation in adults, in line with recent findings (Chen et al., 2015, Plichta et al., 2011, Pollonini et al., 2014, Sevy et al., 2010). Additional, although less consistent, activation was observed over left frontal/motor areas: regions commonly implicated in wider speech processing networks (Friederici, 2011). We did not detect any statistically significant activation in response to visual-only speech, although the channels that came closest to reaching significance were similarly located to the channels that were activated by auditory-only and audiovisual speech (Fig. 3).

**Fig. 4:**
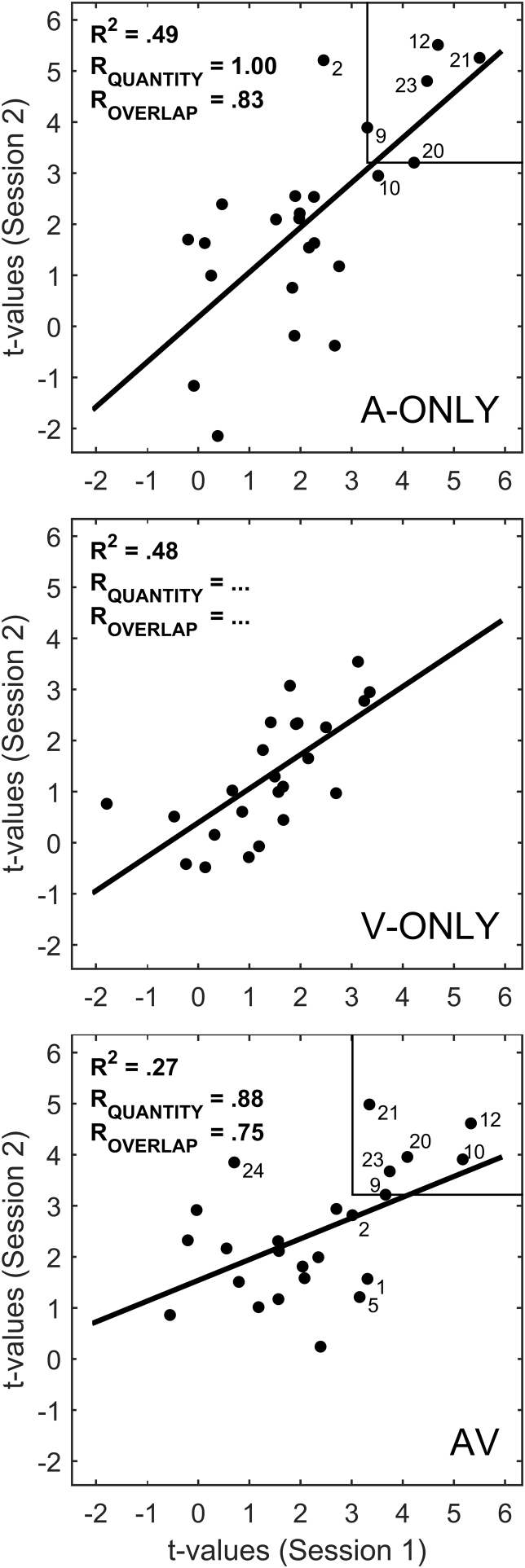
Scatter plots of the *t*-values from the group-level analysis. Results for each stimulation condition are shown in a separate panel. Channels that showed significant activation at either session are labelled with the channel number. A rectangular region in the upper right corner of each panel demarcates channels that were commonly activated at both sessions (if any). Relevant statistics are shown in the upper left corner of each panel. This figure is for the estimated functional component of the haemodynamic response; for corresponding figures for HbO and HbR assessed separately, see Supplementary Figs. S4 and S5.

Reproducibility of group-level activation patterns across the entire probe set was fair (*r* = 0.52, AV condition) to good (*r* = 0.70, A-ONLY condition; *r* = 0.69, V-ONLY condition), as assessed by the linear correlation between individual-channel *t*-values at sessions 1 and 2 (Fig. 4 and Table 1). Note that this metric is influenced by all channels, regardless of whether they overlaid areas of significant cortical activation or not. Taking a more focused view, reproducibility in both the quantity and location of significantly activated channels was excellent for auditory-only (*R*_QUANTITY_ = 1.00, *R*_OVERLAP_ = 0.83) and audiovisual (*R*_QUANTITY_ = 0.88, *R*_OVERLAP_ = 0.75) speech. Since we did not observe any significantly activated channels at the group level in response to visual speech, *R*_QUANTITY_ and *R*_OVERLAP_ could not be calculated for this condition.

Estimating the functional component of the haemodynamic response (Yamada et al., 2012) was critical to our ability to reliably detect significant group-level activation to auditory-only and audiovisual speech (Table 1). When assessing HbO and HbR separately, a conventional approach taken in a majority of fNIRS studies, we found reproducibility to be markedly poorer. Indeed, in the case of HbO, we detected little significant activation at all.

#### 3.2.2 Single-subject level

Consistent with previous studies (Blasi et al., 2014, Plichta et al., 2006, Schecklmann et al., 2008), we found the reproducibility of single-subject cortical activation patterns measured using fNIRS to be highly variable (Table 2). While for some individuals all the reported metrics suggested excellent reproducibility, for other individuals reproducibility across sessions was poor. On average, reproducibility of the quantity of significantly activated channels at single-subject level was good regardless of the mode of speech presentation (*R*_QUANTITY_ = 0.68–0.72 for the estimated functional component), although reproducibility of the location of significant activations was only fair (*R*_OVERLAP_ = 0.41–0.56). Average reproducibility across the entire probe set was fair at best (*r* = 0.32–0.46).

Reproducibility in the quantity and location of significantly activated channels at single-subject level was poorer for HbO than for either HbR or the estimated functional component (Table 2). However, there was no clear evidence that estimating the functional component of the haemodynamic response (Yamada et al., 2012) could significantly improve reproducibility at single-subject level, compared to assessing HbR alone. These observations were confirmed through statistical analyses using the linear mixed model approach (West et al., 2006). Models included fixed effects for stimulation condition (A-ONLY/V-ONLY/AV), haemodynamic measure (estimated functional component/HbO/HbR), plus the stimulation condition x haemodynamic measure interaction, and a random intercept for each individual. There was a significant main effect of haemodynamic measure for both *R*_QUANTITY_ (*F*(2, 128) = 20.82, *p* < 0.001) and *R*_OVERLAP_ (*F*(2, 128) = 16.40, *p* < 0.001). For both metrics, Bonferroni-corrected pairwise comparisons of the estimated marginal means confirmed that reproducibility was significantly poorer for HbO than for either HbR or the estimated functional component (*p* < 0.001 in all cases), but HbR and the estimated functional component did not differ significantly (*p* = 1.00 for both metrics).

**Fig. 5:**
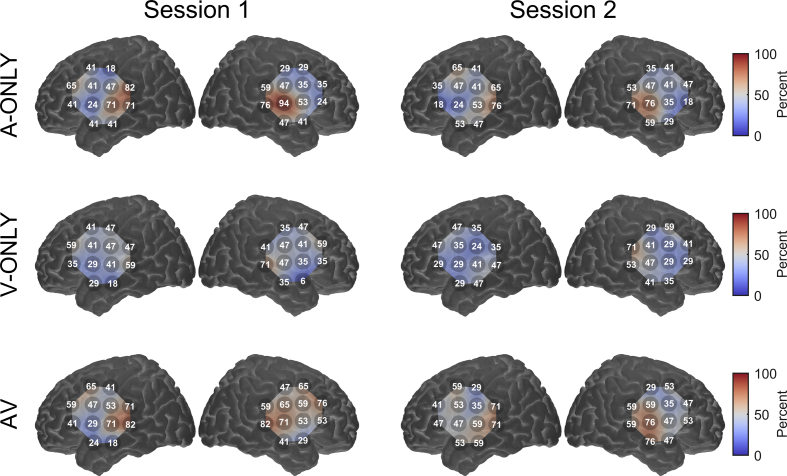
Percentage of individual participants who showed significant activation in each channel (*p* < 0.05 after false discovery rate correction). Results for each stimulation condition are shown in a separate row. This figure is for the estimated functional component of the haemodynamic response; for corresponding figures for HbO and HbR assessed separately, see Supplementary Figs. S6 and S7.

We assessed the spatial distribution of significant activations at single-subject level by plotting the percentage of participants who showed significant activation (*p* < 0.05 after FDR correction) on a channel-wise basis (Fig. 5). For the A-ONLY and AV conditions especially, the majority of significant activations fell within the predefined ROIs. This suggests that these activations likely reflected genuine stimulus-driven cortical activity. However, most channels, including ones well outside the ROIs, were still reported as being significantly activated in around one-third to one-half of individuals. This could reflect genuine dispersed and individually variable cortical activation in wider speech processing networks (FitzGerald et al., 1997, Hall et al., 2005). Alternatively, it could suggest a possible susceptibility to false-positive activations when assessing fNIRS data at single-subject level, even after correcting for serial correlation and multiple comparisons. Further research is needed to quantify the sensitivity and specificity of fNIRS for detecting speech-evoked cortical responses in individual listeners.

### 3.3 Reliability of cortical responses within the ROIs

#### 3.3.1 Time course of haemoglobin concentration changes

**Fig. S11:**
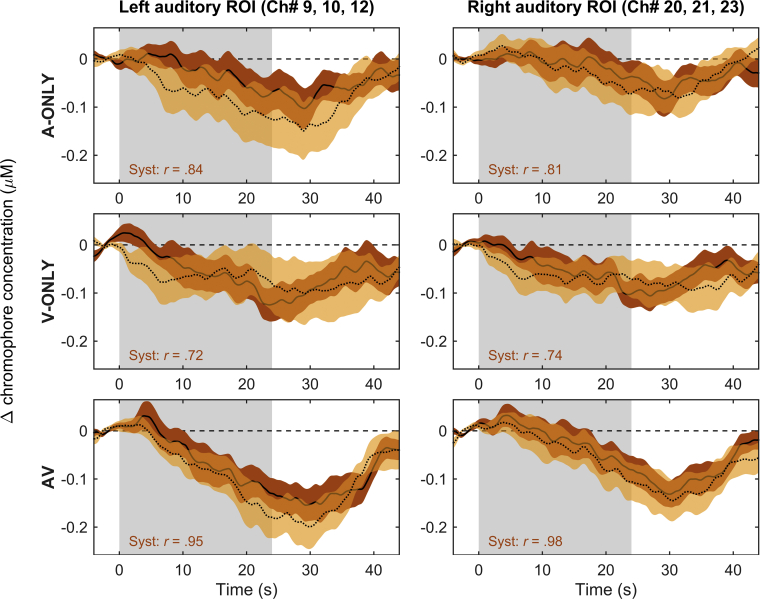
Grand-average time courses for the estimated systemic component of the haemodynamic signal within the predefined ROIs for session 1 (solid lines) and session 2 (dotted lines). Shading is used to indicate ±1 standard error of the mean across participants.

**Fig. 6:**
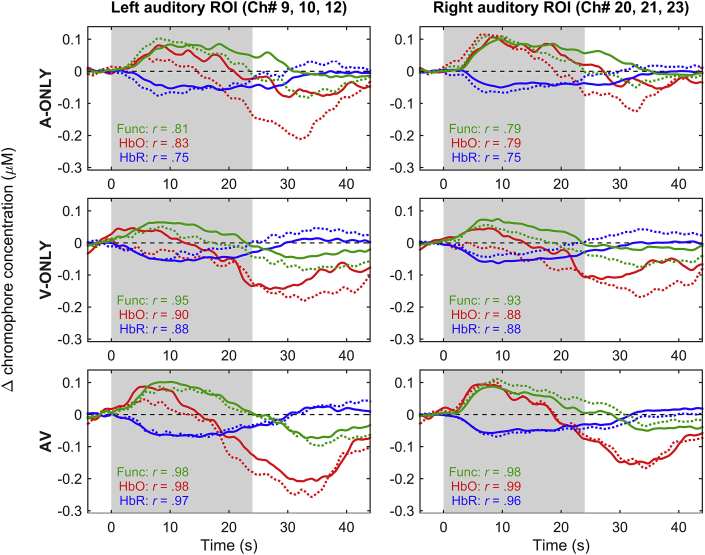
Grand-average time courses within the predefined ROIs for session 1 (solid lines) and session 2 (dotted lines). Results for each stimulation condition are shown in a separate row. Data are plotted for the estimated functional component of the haemodynamic response (green), and for HbO (red) and HbR (blue) assessed separately. Pearson correlation coefficients between session 1 and session 2 time courses are shown in the lower left corner of each panel. The shaded grey areas indicate the stimulation period. See Supplementary Figs. S8–S10 for versions of this figure showing across-subject measurement variance for the estimated functional component, HbO and HbR, respectively. (For interpretation of the references to colour in this figure legend, the reader is referred to the web version of this article.)

At group level, reproducibility in the shape of the haemodynamic time course (block-averaged across the five repetitions of each stimulation condition) was excellent regardless of the mode of speech presentation (Fig. 6, *r* = 0.79–0.98 for the between-session correlations based on the estimated functional component). Reproducibility was generally excellent for HbO and HbR also (*r* ≥ 0.75). However, the HbO time courses exhibited features that appear consistent with this chromophore having greater susceptibility to interference from systemic physiological signals of extra-cerebral origin (Franceschini et al., 2003, Kirilina et al., 2013). Specifically, the HbO time courses generally showed i) a response that was not sustained for the duration of stimulation, and ii) pronounced excursions outside of the stimulation period. Nonetheless, the fact that the HbO time courses were still highly reproducible across sessions (*r* = 0.79–0.99) suggests that even extra-cerebral contributions to the measured signal may have been time-locked to the stimulation cycle (cf. Kirilina et al., 2012). In support of this, when we examined the estimated systemic component of the haemodynamic signal, instead of the functional component as considered elsewhere in the manuscript, we found that the systemic component was itself highly reproducible across sessions (*r* = 0.72–0.98). The systemic component suggested a gradual reduction in superficial haemoglobin concentrations that began shortly after the onset of stimulation and did not reach a minimum until several seconds after the offset of stimulation (Supplementary Fig. S11).

**Fig. S12:**
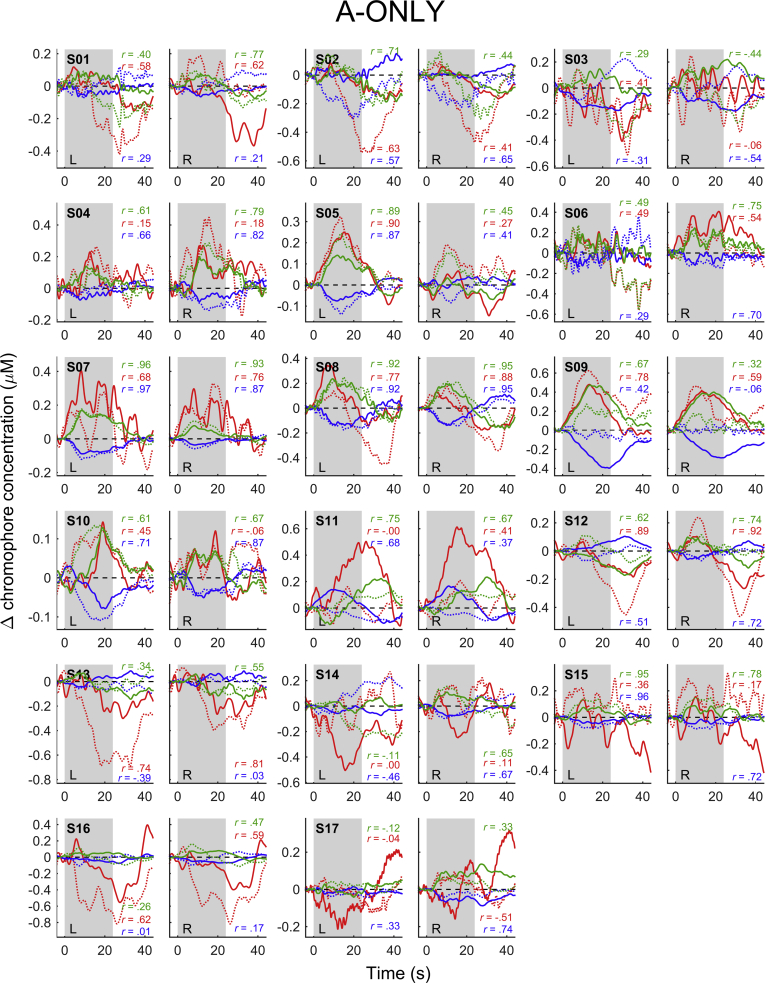
Individual time courses for all 17 participants (S01–S17) for the A-ONLY condition (solid lines = session 1, dotted lines = session 2; green = estimated functional component, red = HbO, blue = HbR).

**Fig. S13:**
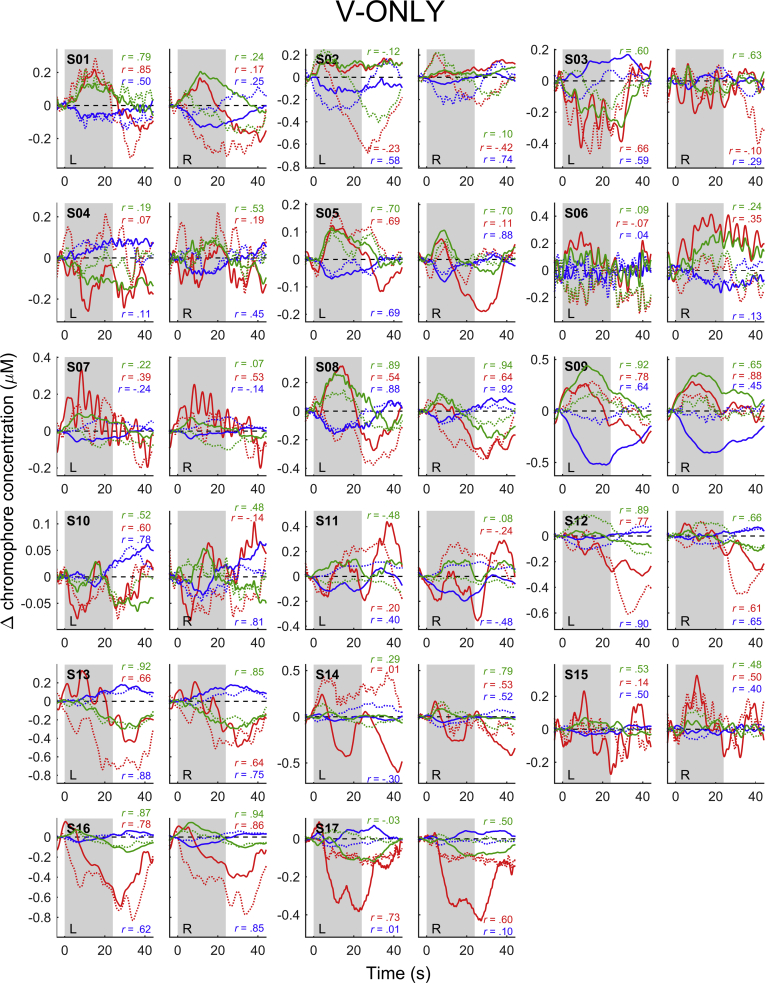
Individual time courses for all 17 participants (S01–S17) for the V-ONLY condition (solid lines = session 1, dotted lines = session 2; green = estimated functional component, red = HbO, blue = HbR).

**Fig. S14:**
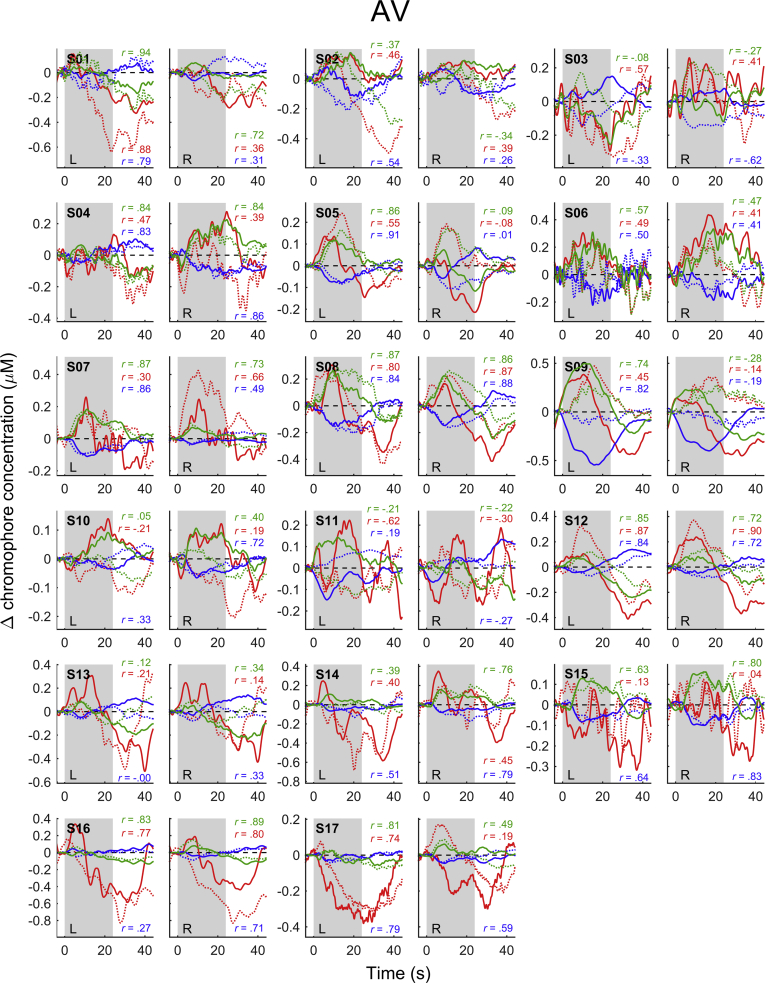
Individual time courses for all 17 participants (S01–S17) for the AV condition (solid lines = session 1, dotted lines = session 2; green = estimated functional component, red = HbO, blue = HbR).

Consistent with previous studies (Blasi et al., 2014, Kakimoto et al., 2009, Kono et al., 2007), we found the reproducibility of single-subject haemodynamic time courses to be highly variable (see Supplementary Figs. S12–S14). Between-session correlation coefficients varied widely across individuals and conditions, taking values between −0.62 and +0.97 in the extreme cases. On average, reproducibility in single-subject haemodynamic time courses was fair (*r* ≈ 0.4–0.5). Collapsed across the left- and right-hemisphere ROIs, mean correlations were consistently highest for the estimated functional component and lowest for HbO, with intermediate values for HbR. However, statistical analysis based on a linear mixed model did not confirm any significant difference between the three haemodynamic measures (main effect of haemodynamic measure: *F*(2, 272) = 2.64, *p* = 0.07).

#### 3.3.2 Reliability of response amplitude

Reliability of response amplitude within the ROIs was assessed using the ICC (Table 3). The results exhibit overall patterns that are consistent with the findings of previous studies (Plichta et al., 2006, Plichta et al., 2007b, Schecklmann et al., 2008). Firstly, values of ICC_2_SESS_AVG_ were typically around 30% higher than corresponding values of ICC_SINGLE_SESS_ (both at single-channel and cluster level). This demonstrates that mean fNIRS responses across two sessions are more reliable than those measured at a single session. Secondly, cluster-level ICCs were on average around 20% higher than the mean of the ICCs for the constituent channels. This confirms that reliability can be improved by averaging across a small number of channels that overlie a cortical ROI.

Based on the ICCs for the estimated functional component, the reliability of responses to auditory speech (with or without visual cues) was fair when assessed at single-channel and single-session level (ICC_SINGLE_SESS_ = 0.42–0.59, mean of single-channel ICCs in the A-ONLY and AV conditions). Reliability was improved by assessing responses at cluster level, and by considering the average response across the two sessions: good reliability was achieved in the right-hemisphere ROI (ICC_2_SESS_AVG_ = 0.68–0.73), and excellent reliability in the left-hemisphere ROI (ICC_2_SESS_AVG_ = 0.77–0.85). Responses to purely visual speech were less reliable, especially in the right-hemisphere ROI where reliability was consistently poor (ICCs < 0.40); however, left-hemisphere responses to visual speech showed fair to good reliability when assessed at cluster level (ICC_SINGLE_SESS_ = 0.56, ICC_2_SESS_AVG_ = 0.71). Generally better reliability of response amplitude in the left-versus the right-hemisphere ROI may reflect left-hemispheric dominance for the processing of connected speech (Peelle, 2012).

Estimating the functional component of the haemodynamic response (Yamada et al., 2012) consistently led to higher reliability, compared to assessing HbO and HbR separately. Indeed, at cluster level, only 1 out of 12 ICCs for each of HbO and HbR indicated good reliability. In contrast, 7 out of 12 cluster-level ICCs for the estimated functional component indicated good or better reliability, with 2 of these indicating excellent reliability.

We additionally tested for any significant group-level differences in mean response amplitude between sessions (Table 4). There was no evidence that response amplitude either consistently increased or decreased from session 1 to session 2. Indeed, none of the paired *t*-tests came close to the threshold for statistical significance (*p* < 0.05), even when uncorrected for multiple comparisons (a conservative approach in this context given our interest in confirming the null hypothesis). Furthermore, the effect size estimates indicated that the effect of session was in most cases negligible (*d* < 0.2).

### 3.4 General discussion

This study aimed to establish the test-retest reliability of speech-evoked fNIRS responses in adults. The basis of any such reliability assessment is the assumption that the underlying physiological processes remain stable over the time period in question: in the present case, a test-retest interval of 3 months. Since none of our participants reported any change in hearing or visual acuity during the study, and speech intelligibility was similar across the two sessions (albeit at ceiling level in the case of auditory and audiovisual speech), we assume that speech-evoked cortical activity was generally stable also. In support of this, we did not find any evidence for a systematic difference in response amplitude between sessions. However, we cannot exclude the possibility that increased familiarity with the test procedure and speech materials at session 2 compared to session 1 may have influenced our results. Indeed, Schecklmann et al. (2008) highlighted that fNIRS test-retest reliability may be affected by variations in both physiological (e.g. stress, arousal) and psychological (e.g. learning, strategy, efficiency) factors across sessions, depending on the time interval in question. In this respect, one limitation of the present study is that measurements were made at only two time points: inclusion of additional time points, including ideally a within-session control based on repeated administration of the paradigm during the initial visit, would have provided a stronger baseline against which to compare test-retest reliability over the longer term.

We found that fNIRS measurements were more reliable: i) at group level than at single-subject level; and ii) when averaged across a small number of channels overlying a cortical ROI than when assessed on a single-channel basis. These have emerged as consistent findings from previous fNIRS test-retest studies (Blasi et al., 2014, Kakimoto et al., 2009, Kono et al., 2007, Plichta et al., 2006, Plichta et al., 2007b, Sato et al., 2006, Schecklmann et al., 2008, Strangman et al., 2006, Watanabe et al., 2003), which have involved a variety of imaging paradigms (block-design versus event-related), brain regions (visual, motor, frontal, and temporal cortices), tasks (from low-level sensory stimulation to higher-level cognitive tasks), and subject populations (varying in age and clinical status).

In the present study, no significantly activated channels were detected at the group level in response to visual speech (after FDR correction). This meant that it was not possible to assess the reproducibility of the quantity (*R*_QUANTITY_) and location (*R*_OVERLAP_) of significantly activated channels for this condition at the group level. Evidence from a previous fMRI study corroborates this absence of significant group-level activation in auditory brain regions during silent speechreading, despite significant activation within individuals (Hall et al., 2005). This apparent discrepancy between single-subject and group-level activation patterns may be due to variability in the cortical networks that individual subjects recruit to process visual linguistic cues. It should be noted, though, that poorer reproducibility of responses to visual speech compared with auditory or audiovisual speech was a consistent finding in the present study, including at single-subject level. This may reflect the fact that responses in the temporal brain regions studied here were significantly weaker when the presented speech did not include an auditory element, potentially resulting in a greater impact of physiological and/or instrumentation noise. Alternatively, because participants found silent speechreading highly challenging and could understand at best only a few words, test-retest reliability in this condition may have been more susceptible to influence from within-subject variations in motivation, cognitive load or psycholinguistic strategy across sessions.

Previous studies of fNIRS test-retest reliability have obtained mixed results regarding the relative merits of HbO and HbR. Several studies reported that HbO had greater power to detect significant activation (Blasi et al., 2014, Kono et al., 2007, Plichta et al., 2006), although this did not always translate to better test-retest reliability. The apparently lower statistical power of HbR has been interpreted as reflecting greater local specificity for this chromophore (Plichta et al., 2006, Plichta et al., 2007b). Interestingly, in the present study, we found HbR to have greater power to detect significant activation than HbO, and activation patterns were also more reproducible for HbR. One possible reason for this is our use of a canonical HRF borrowed from fMRI analysis, since the blood-oxygen-level dependent (BOLD) signal measured in fMRI is physiologically more closely related to the HbR signal (Buxton et al., 1998). Indeed, the BOLD response has been found to be temporally more highly correlated with HbR than with HbO (Huppert et al., 2006). However, subtle timing differences between HbO and HbR would generally not be expected to have a major influence in the case of a block-design paradigm as used here, on the basis that the predicted haemodynamic time course will quickly reach a steady-state plateau during blocked stimulation, regardless of slight differences in the transition times at the onset and offset of stimulation (Huppert et al., 2006). Rather, we believe that the poorer sensitivity of HbO to reliably detect significant cortical activation in the present study likely reflects greater contamination of the HbO signal by physiological noise of extra-cerebral origin (see Fig. 6 and associated discussion).

Regarding the comparative reliability of our fNIRS measurements, when HbO and HbR were assessed separately, we found the reliability of speech-evoked activation in temporal cortex to be somewhat lower than has been reported for other brain regions and tasks (cf. Plichta et al., 2006, Plichta et al., 2007b, Schecklmann et al., 2008). We are not certain why this should be the case. One possibility is that fNIRS measurements have lower sensitivity to auditory cortical regions than to other brain regions. This is almost certainly true for the primary auditory cortex, which, lying in the depths of the lateral sulcus (Penhune et al., 1996), is likely too deep to be sampled effectively by depth-limited fNIRS measurements (Strangman et al., 2013). Auditory association areas, important for speech processing and located more laterally (Friederici, 2011), are in principle more accessible, although regional differences in scalp and skull thickness can still affect the sensitivity of fNIRS to grey matter (Strangman et al., 2014). Unfortunately, numerous differences between studies (in imaging paradigm, measurement duration, retest interval, task demands, test population, and so on), make it difficult to draw firm conclusions regarding the relative reliability of fNIRS measurements across different brain regions.

Despite the reliability issues that we observed when assessing HbO and HbR separately, by combining the two chromophores to estimate the functional component of the haemodynamic response (Yamada et al., 2012), we were able to achieve group-level reliability metrics that were comparable to best-case values reported in the literature for other brain regions and tasks (Kono et al., 2007, Plichta et al., 2006, Plichta et al., 2007b, Schecklmann et al., 2008). To our knowledge, this is the first time this type of physiological noise reduction algorithm has been evaluated in a study of fNIRS test-retest reliability. However, Biallas et al. (2012) did assess an alternative technique for reducing the influence of systemic physiological signals, which is to include a reference channel with short inter-optode spacing. This allows signals that originate in superficial tissue layers to be regressed out from the main measurement channels (Gagnon et al., 2011). Biallas et al. (2012) found that inclusion of the short-separation reference channel increased the sensitivity of fNIRS for detecting visually evoked cortical responses from 36.8% to 55.2%. We conclude that any approach that is effective in reducing the influence of systemic physiological interference is likely to improve the reliability of fNIRS measurements.

As previous studies have noted (e.g. Plichta et al., 2007b), the positioning of optodes with respect to surface landmarks (as performed here) may have a bearing on fNIRS test-retest reliability: variation in optode placement across sessions may reduce reliability at the single-subject level, while variability in cranio-cerebral relationships among individuals may limit the reliability of group-level analyses. Interestingly, our ICC analysis showed that reliability was consistently improved by taking the average response across the two sessions, suggesting that inconsistency in optode placement across sessions is unlikely to have been a major issue here. As for individual variability in cranio-cerebral relationships, we anticipate that advances in optical imaging technology that allow three-dimensional reconstruction of activity within an anatomically accurate brain model will bring further improvements in reliability (see, for example, Hassanpour et al., 2015).

## 4 Conclusions

We conclude that fNIRS is capable of measuring temporal-lobe responses to auditory speech (with or without visual cues) that have good-to-excellent test-retest reliability at the group level. Thus, fNIRS holds promise as a silent, flexible, and non-invasive tool for studying central auditory processing. On average, reliability at the single-subject level was fair, with wide variation across individuals. Further refinements are therefore needed before reliable measurements can be guaranteed at the individual level. We offer the following advice to researchers conducting auditory fNIRS studies and wishing to maximize the reliability of their measurements: i) Use a proven strategy to reduce the influence of systemic physiological noise; ii) Consider averaging across a small number of channels that overlie a cortical ROI when assessing response amplitude; iii) If it is not possible to collect sufficient data within a single imaging session, measurement reliability may be improved by averaging each individual's data across multiple sessions, providing sufficient care is taken to maintain consistent optode positioning.

## Figures and Tables

**Fig. S2:**
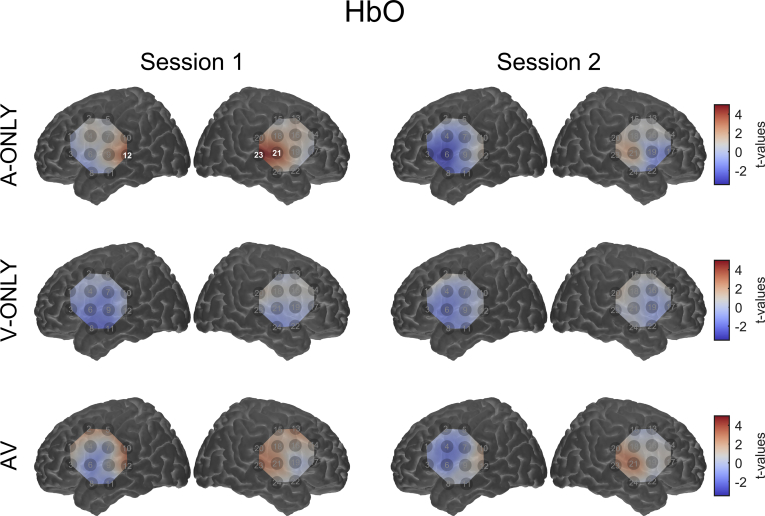
Group-level activation maps for HbO. Channels that showed significant activation are highlighted (*p* < .05 after false discovery rate correction).

**Fig. S3:**
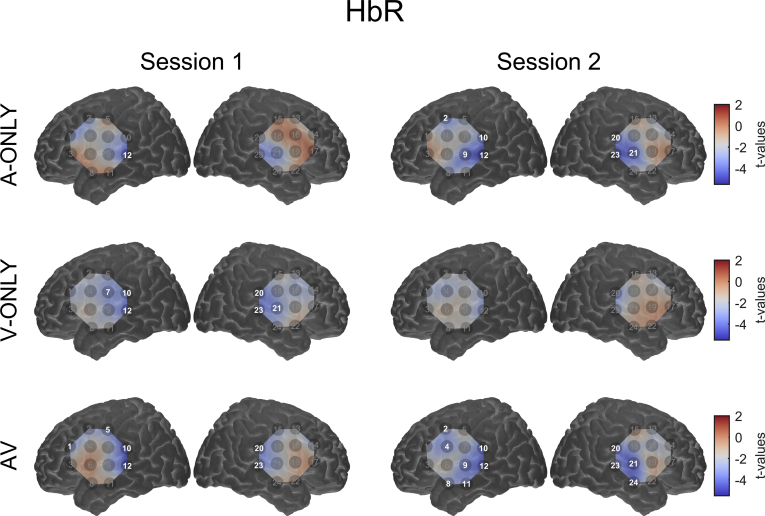
Group-level activation maps for HbR. Channels that showed significant activation are highlighted (*p* < .05 after false discovery rate correction).

**Fig. S4:**
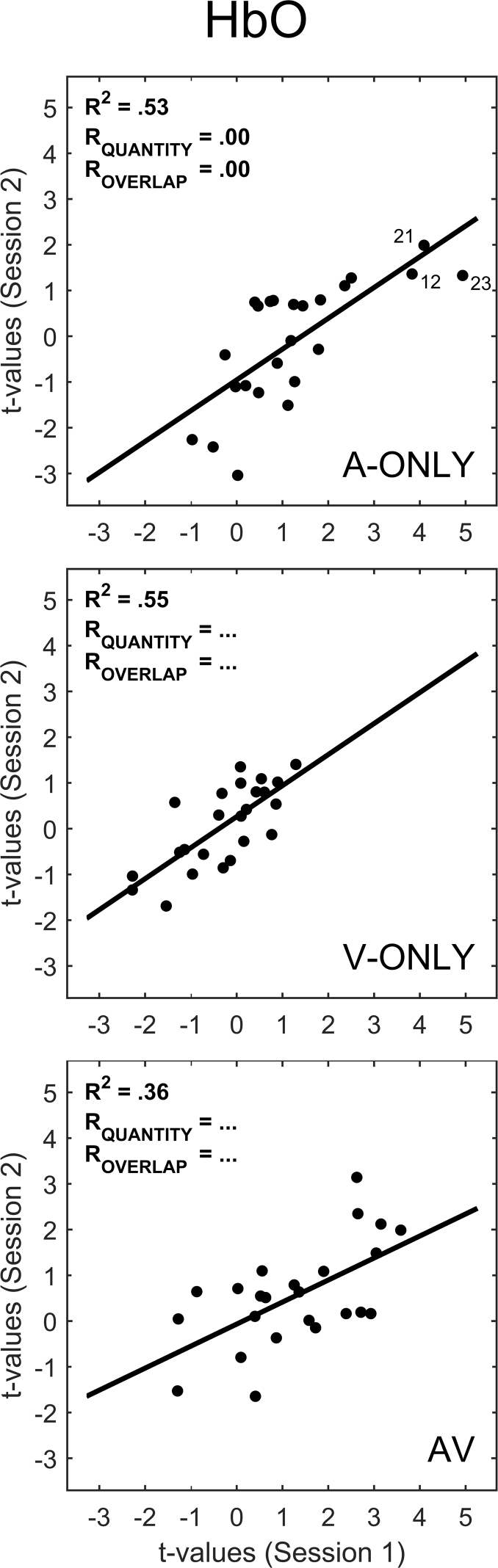
Group-level *t*-value scatter plots for HbO.

**Fig. S5:**
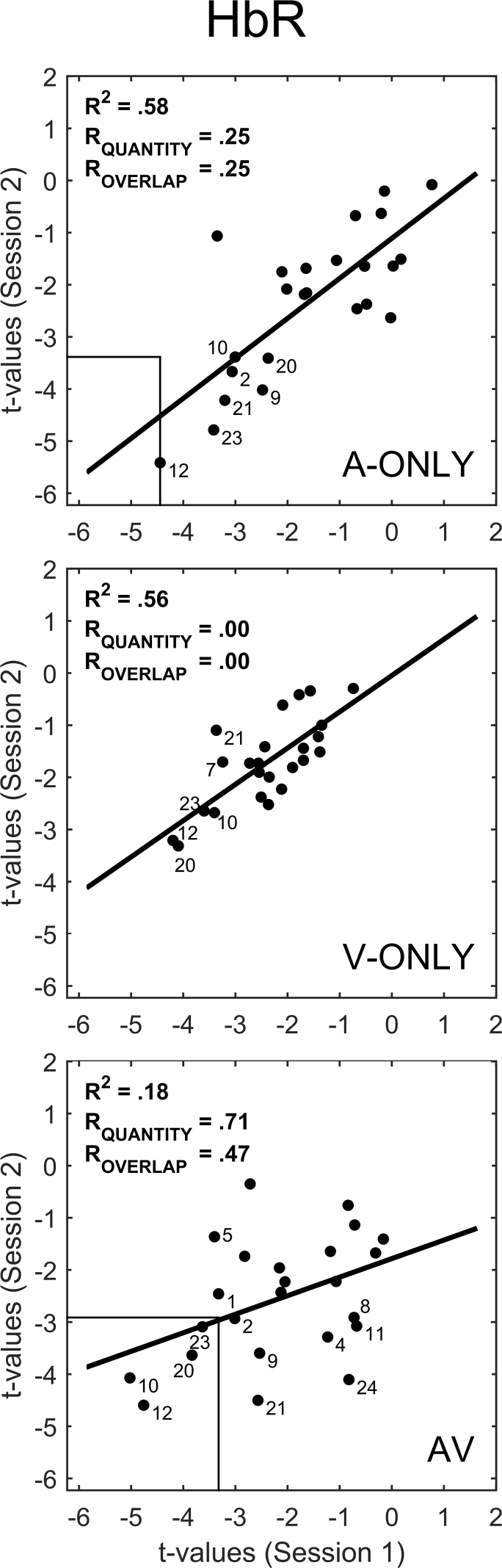
Group-level *t*-value scatter plots for HbR.

**Fig. S6:**
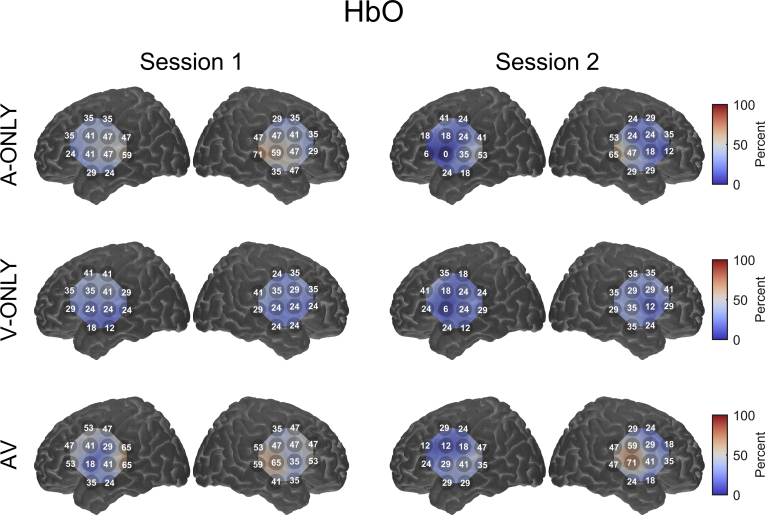
Percentage of individuals who showed significant activation in each channel for HbO (*p* < .05 after false discovery rate correction).

**Fig. S7:**
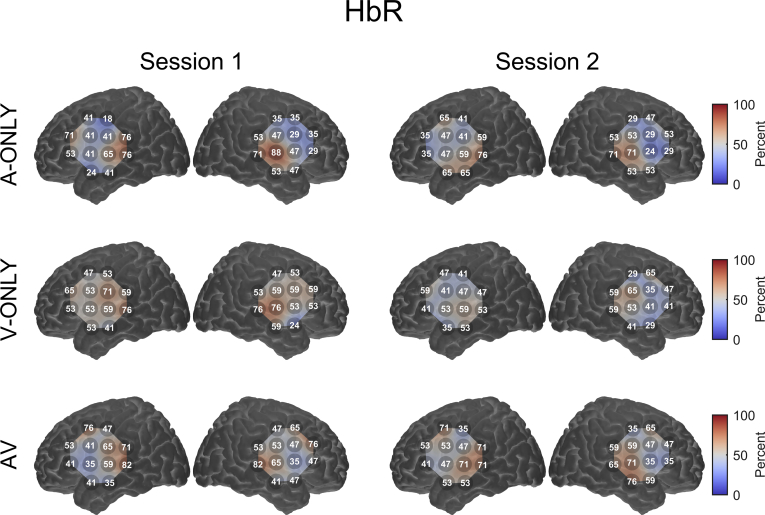
Percentage of individuals who showed significant activation in each channel for HbR (*p* < .05 after false discovery rate correction).

**Fig. S8:**
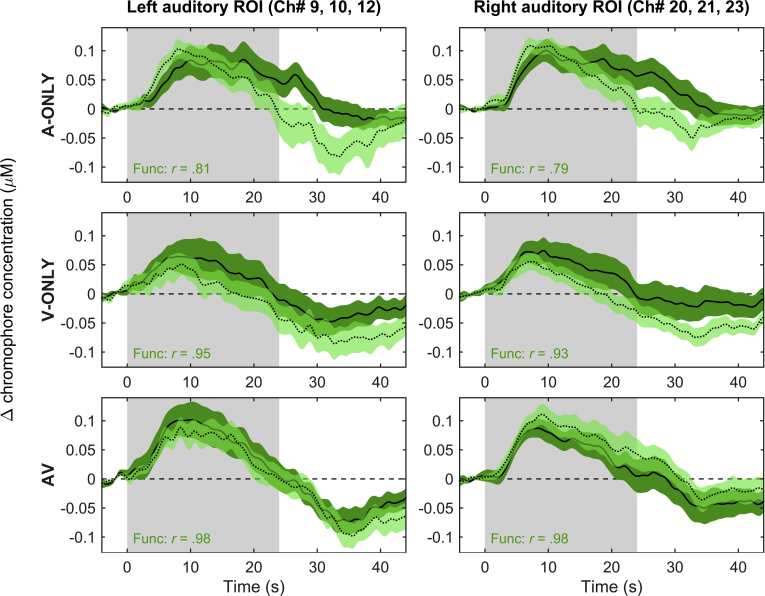
Grand-average time courses for the estimated functional component within the predefined ROIs for session 1 (solid lines) and session 2 (dotted lines). Shading is used to indicate ±1 standard error of the mean across participants.

**Fig. S9:**
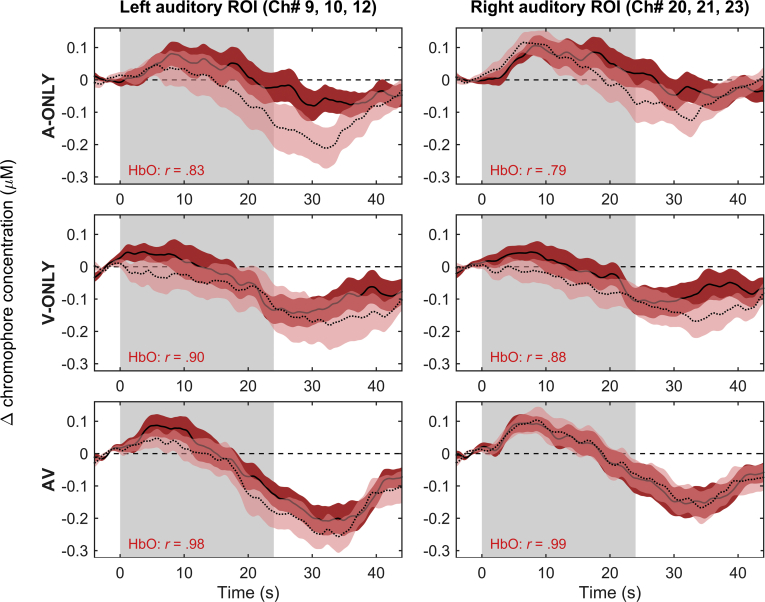
Grand-average time courses for HbO within the predefined ROIs for session 1 (solid lines) and session 2 (dotted lines). Shading is used to indicate ±1 standard error of the mean across participants.

**Fig. S10:**
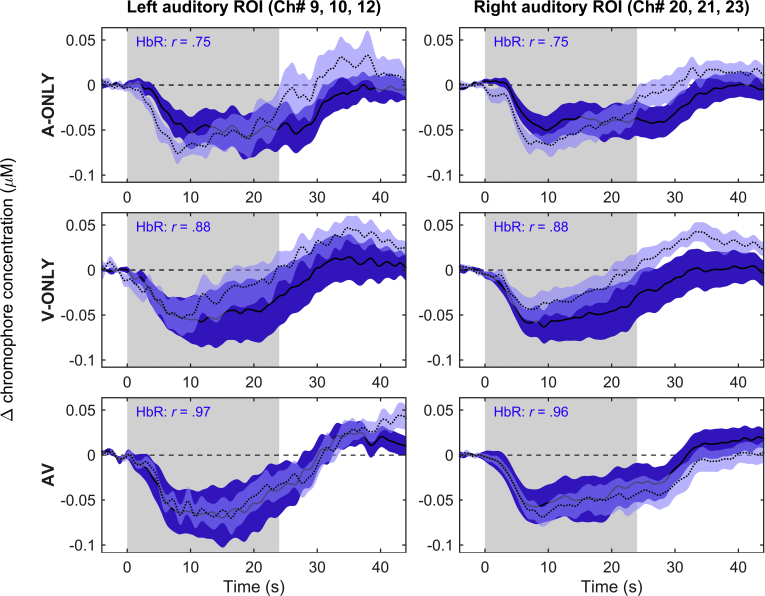
Grand-average time courses for HbR within the predefined ROIs for session 1 (solid lines) and session 2 (dotted lines). Shading is used to indicate ±1 standard error of the mean across participants.

**Table 1:** Number of significantly activated channels (*p* < 0.05 after false discovery rate correction) and reproducibility metrics for the group-level activation maps.

	No. of significantly activated channels^a^		Reproducibility metrics^b^		
	Session 1	Session 2	*r*	*R*_QUANTITY_	*R*_OVERLAP_
**A-ONLY**					
Functional	6 (3L, 3R)	6 (3L, 3R)	0.70	1.00	0.83
HbO	3 (1L, 2R)	0 (0L, 0R)	0.73	0.00	0.00
HbR	1 (1L, 0R)	7 (4L, 3R)	0.76	0.25	0.25
**V-ONLY**					
Functional	0 (0L, 0R)	0 (0L, 0R)	0.69	–	–
HbO	0 (0L, 0R)	0 (0L, 0R)	0.74	–	–
HbR	6 (3L, 3R)	0 (0L, 0R)	0.75	0.00	0.00
**AV**					
Functional	9 (6L, 3R)	7 (3L, 4R)	0.52	0.88	0.75
HbO	0 (0L, 0R)	0 (0L, 0R)	0.60	–	–
HbR	6 (4L, 2R)	11 (7L, 4R)	0.42	0.71	0.47

a Values in parentheses give the number of significantly activated channels in each hemisphere.

b Missing values indicate that there were no significantly activated channels at either session.

**Table 2:** Reproducibility metrics for single-subject activation maps. Missing values indicate that there were no significantly activated channels at either session.

Participant	Functional			HbO			HbR		
	*r*	*R*_QUANTITY_	*R*_OVERLAP_	*r*	*R*_QUANTITY_	*R*_OVERLAP_	*r*	*R*_QUANTITY_	*R*_OVERLAP_
**A-ONLY**									
1	0.44	0.00	0.00	0.22	0.60	0.40	0.39	0.17	0.00
2	0.16	0.40	0.30	−0.36	0.00	0.00	0.52	0.67	0.60
3	−0.12	0.85	0.61	−0.67	0.36	0.18	−0.26	0.90	0.55
4	0.26	0.95	0.53	0.18	0.17	0.17	0.07	0.55	0.36
5	0.82	0.91	0.73	0.80	0.80	0.70	0.74	0.74	0.63
6	0.34	0.77	0.31	0.50	0.94	0.47	0.12	0.71	0.29
7	0.80	0.93	0.80	−0.13	0.00	0.00	0.73	0.70	0.60
8	0.66	0.84	0.84	0.66	0.40	0.40	0.66	0.95	0.90
9	0.30	0.69	0.69	0.42	0.74	0.74	0.39	0.58	0.58
10	0.49	0.57	0.57	0.50	0.71	0.57	0.37	0.64	0.64
11	0.22	0.73	0.36	0.11	0.78	0.22	0.02	0.75	0.25
12	0.40	0.86	0.81	0.49	0.86	0.86	0.41	1.00	0.83
13	0.42	0.00	0.00	0.53	0.00	0.00	0.40	0.31	0.31
14	0.39	0.95	0.57	0.49	0.00	0.00	0.24	1.00	0.50
15	0.86	0.69	0.69	0.55	0.37	0.37	0.82	0.54	0.54
16	0.80	0.97	0.91	0.36	0.00	0.00	0.41	0.98	0.93
17	0.65	0.77	0.62	0.66	0.00	0.00	0.71	0.88	0.63
Mean	0.46	0.70	0.55	0.31	0.40	0.30	0.40	0.71	0.54
**V-ONLY**									
1	−0.59	0.38	0.31	−0.15	0.69	0.63	−0.54	0.24	0.16
2	0.48	0.67	0.22	0.79	0.00	0.00	0.62	0.87	0.82
3	0.17	0.67	0.00	0.75	0.00	0.00	0.16	0.50	0.00
4	0.55	1.00	0.00	0.01	0.00	0.00	0.67	0.29	0.29
5	0.50	0.97	0.76	0.68	0.96	0.80	0.28	0.91	0.79
6	0.50	0.76	0.62	0.46	0.92	0.58	0.33	0.88	0.69
7	0.41	0.63	0.50	−0.17	0.29	0.00	0.55	0.59	0.59
8	0.24	0.71	0.65	0.48	0.00	0.00	−0.12	0.92	0.76
9	−0.52	0.67	0.40	−0.59	0.96	0.24	−0.27	0.58	0.52
10	0.18	0.67	0.33	−0.11	0.00	0.00	0.46	0.82	0.55
11	0.35	0.50	0.00	0.25	0.00	0.00	−0.01	0.00	0.00
12	0.68	0.80	0.72	0.26	0.52	0.52	0.76	0.91	0.73
13	0.37	0.08	0.08	0.05	0.08	0.08	0.15	0.00	0.00
14	0.70	0.80	0.72	0.34	0.40	0.00	0.68	0.85	0.77
15	0.47	0.97	0.69	0.49	0.14	0.14	0.40	0.97	0.69
16	0.66	0.87	0.73	0.35	–	–	0.54	0.80	0.73
17	0.36	0.50	0.17	0.38	–	–	0.46	0.74	0.67
Mean	0.32	0.68	0.41	0.25	0.33	0.20	0.30	0.64	0.51
**AV**									
1	−0.14	0.63	0.21	0.53	0.00	0.00	−0.27	0.61	0.26
2	0.64	0.81	0.74	0.70	0.87	0.80	0.06	0.00	0.00
3	−0.21	0.27	0.27	0.59	0.80	0.60	−0.18	0.11	0.11
4	0.38	0.97	0.73	0.57	0.88	0.75	0.06	1.00	0.67
5	0.17	0.43	0.43	0.28	0.35	0.24	0.31	0.64	0.64
6	0.37	0.52	0.43	0.59	0.40	0.40	0.31	0.64	0.56
7	0.66	0.76	0.48	0.18	0.52	0.52	0.63	0.90	0.60
8	0.62	1.00	0.91	0.72	0.72	0.72	0.66	0.95	0.91
9	0.15	0.85	0.80	−0.04	0.67	0.44	0.13	0.67	0.67
10	0.19	0.60	0.20	−0.17	0.00	0.00	0.06	0.93	0.57
11	0.05	0.89	0.44	−0.30	0.29	0.00	0.52	0.50	0.50
12	0.52	0.86	0.81	0.58	0.93	0.93	0.57	0.79	0.73
13	0.19	0.18	0.18	0.64	0.67	0.67	0.13	0.29	0.14
14	0.21	0.94	0.56	0.12	0.69	0.56	0.31	0.96	0.59
15	0.83	0.95	0.84	0.62	0.00	0.00	0.78	0.98	0.93
16	0.92	0.94	0.94	0.11	0.00	0.00	0.81	0.97	0.92
17	0.62	0.62	0.46	0.60	0.00	0.00	0.71	0.75	0.63
Mean	0.36	0.72	0.56	0.37	0.46	0.39	0.33	0.69	0.55

**Table 3:** Reliability of response amplitude within the predefined ROIs based on the intraclass correlation coefficient (ICC).

		Single channels^a^		Cluster level^b^	
		ICC_SINGLE_SESS_	ICC_2_SESS_AVG_	ICC_SINGLE_SESS_	ICC_2_SESS_AVG_
**A-ONLY**					
Functional	Left	0.59 (0.44–0.70)	0.74 (0.61–0.82)	0.74	0.85
	Right	0.42 (0.41–0.44)	0.60 (0.59–0.61)	0.51	0.68
HbO	Left	0.23 (0.04–0.40)	0.35 (0.07–0.57)	0.25	0.41
	Right	0.10 (0.00–0.27)	0.15 (0.00–0.43)	0.04	0.08
HbR	Left	0.43 (0.04–0.70)	0.54 (0.07–0.82)	0.48	0.65
	Right	0.27 (0.02–0.54)	0.39 (0.04–0.70)	0.18	0.31
**V-ONLY**					
Functional	Left	0.33 (0.18–0.44)	0.49 (0.31–0.61)	0.56	0.71
	Right	0.20 (0.00–0.41)	0.29 (0.00–0.59)	0.06	0.11
HbO	Left	0.22 (0.12–0.40)	0.34 (0.21–0.57)	0.30	0.46
	Right	0.01 (0.00–0.01)	0.02 (0.00–0.03)	0.00	0.00
HbR	Left	0.27 (0.26–0.29)	0.43 (0.42–0.45)	0.33	0.50
	Right	0.06 (0.00–0.18)	0.10 (0.00–0.30)	0.00	0.00
**AV**					
Functional	Left	0.50 (0.31–0.65)	0.66 (0.48–0.79)	0.63	0.77
	Right	0.58 (0.54–0.62)	0.74 (0.70–0.77)	0.58	0.73
HbO	Left	0.34 (0.25–0.51)	0.50 (0.40–0.67)	0.39	0.56
	Right	0.40 (0.32–0.57)	0.57 (0.48–0.73)	0.45	0.62
HbR	Left	0.28 (0.13–0.56)	0.41 (0.23–0.72)	0.33	0.50
	Right	0.38 (0.18–0.63)	0.53 (0.31–0.77)	0.22	0.36

a Values in parentheses give the range of ICCs across individual channels within the ROI.

b Beta weights were averaged across the constituent channels prior to calculating cluster-level ICCs.

**Table 4:** Results of paired *t-*tests comparing mean response amplitude within the predefined ROIs across sessions.

		Mean (SD) ROI beta weight		Direction of change^a^	*p*-value^b^	Effect size (*d*)
		Session 1	Session 2			
**A-ONLY**						
Functional	Left	0.066 (0.059)	0.073 (0.061)	+ve	0.511	0.16
	Right	0.071 (0.040)	0.069 (0.046)	-ve	0.906	0.03
HbO	Left	0.062 (0.078)	0.039 (0.178)	-ve	0.573	0.14
	Right	0.074 (0.067)	0.065 (0.160)	-ve	0.828	0.05
HbR	Left	−0.042 (0.047)	−0.050 (0.037)	+ve	0.490	0.17
	Right	−0.038 (0.042)	−0.043 (0.026)	+ve	0.667	0.11
**V-ONLY**						
Functional	Left	0.036 (0.053)	0.029 (0.048)	-ve	0.567	0.14
	Right	0.035 (0.034)	0.035 (0.049)	+ve	0.954	0.01
HbO	Left	−0.008 (0.135)	0.008 (0.104)	-ve	0.656	0.11
	Right	0.004 (0.098)	0.020 (0.098)	+ve	0.668	0.11
HbR	Left	−0.037 (0.041)	−0.030 (0.047)	-ve	0.592	0.13
	Right	−0.034 (0.033)	−0.030 (0.052)	-ve	0.824	0.05
**AV**						
Functional	Left	0.077 (0.057)	0.068 (0.058)	-ve	0.480	0.18
	Right	0.068 (0.058)	0.074 (0.056)	+ve	0.630	0.12
HbO	Left	0.066 (0.103)	0.040 (0.113)	-ve	0.388	0.22
	Right	0.078 (0.100)	0.074 (0.105)	-ve	0.878	0.04
HbR	Left	−0.051 (0.049)	−0.047 (0.040)	-ve	0.772	0.07
	Right	−0.042 (0.045)	−0.043 (0.035)	+ve	0.984	0.00

a +ve indicates a stronger cortical response at session 2 than at session 1.

b Unadjusted (i.e. without correction for multiple comparisons).
